# Stable luciferase expression does not alter immunologic or *in vivo* growth properties of GL261 murine glioma cells

**DOI:** 10.1186/s12967-014-0345-4

**Published:** 2014-12-03

**Authors:** Aaron J Clark, Michael Safaee, Taemin Oh, Michael E Ivan, Vamsi Parimi, Rintaro Hashizume, Tomoko Ozawa, Charles D James, Orin Bloch, Andrew T Parsa

**Affiliations:** The Brain Tumor Research Center, Department of Neurological Surgery, University of California, San Francisco, CA 505 Parnassus Ave., Room 779 M, San Francisco, CA 94143-0112 USA; Department of Neurological Surgery, Northwestern University, Feinberg School of Medicine, 676 N. St. Clair St., Suite 2210, Chicago, IL 60611-2922 USA; Pathology Core Facility, Feinberg School of Medicine, Northwestern University, 710 N. Fairbanks Court, Room 8-419, Chicago, IL USA; Department of Neurological Surgery, University of California, San Francisco, 505 Parnassus Ave., M779, Box 0112, San Francisco, CA 94117 USA

**Keywords:** Glioma, Luciferase, Mouse model, Immunotherapy, Microenvironment, GL261

## Abstract

**Background:**

GL261 cells are murine glioma cells that demonstrate proliferation, invasion, and angiogenesis when implanted in syngeneic C57BL/6 mice, providing a highly useful immunocompetent animal model of glioblastoma. Modification of tumor cells for luciferase expression enables non-invasive monitoring of orthotopic tumor growth, and has proven useful for studying glioblastoma response to novel therapeutics. However, tumor modification for luciferase has the potential for evoking host immune response against otherwise syngeneic tumor cells, thereby mitigating the tumor cells’ value for tumor immunology and immunotherapy studies.

**Methods:**

GL261 cells were infected with lentivirus containing a gene encoding firefly luciferase (GL261.luc). *In vitro* proliferation of parental (unmodified) GL261 and GL261.luc was measured on days 0, 1, 2, 4, and 7 following plating, and the expression of 82 mouse cytokines and chemokines were analyzed by RT-PCR array. Cell lines were also evaluated for differences in invasion and migration in modified Boyden chambers. GL261 and GL261.luc cells were then implanted intracranially in C57BL/6 mice, with GL261.luc tumor growth monitored by quantitative bioluminescence imaging, and all mice were followed for survival to compare relative malignancy of tumor cells.

**Results:**

No difference in proliferation was indicated for GL261 vs. GL261.luc cells (p>0.05). Of the 82 genes examined by RT-PCR array, seven (9%) exhibited statistically significant change after luciferase modification. Of these, only three changed by greater than 2-fold: BMP-2, IL-13, and TGF-β2. No difference in invasion (p=0.67) or migration (p=0.26) was evident between modified vs. unmodified cells. GL261.luc cell luminescence was detectable in the brains of C57BL/6 mice at day 5 post-implantation, and tumor bioluminescence increased exponentially to day 19. Median overall survival was 20.2 days versus 19.7 days for mice receiving implantation with GL261 and GL261.luc, respectively (p=0.62). Histopathologic analysis revealed no morphological difference between tumors, and immunohistochemical analysis showed no significant difference for staining of CD3, Ki67, or CD31 (p>0.05 for all).

**Conclusions:**

Luciferase expression in GL261 murine glioma cells does not affect GL261 proliferation, invasion, cytokine expression, or *in vivo* growth. Luciferase modification increases their utility for studying tumor immunology and immunotherapeutic approaches for treating glioblastoma.

## Introduction

A primary goal of glioblastoma (GBM) immunotherapy is to harness the body’s immune system to target and eradicate tumor cells, but the immunosuppressive microenvironment in GBM has proven to significantly limit the efficacy of immunotherapeutic strategies [1]. Preclinical animal models of GBM are critical to the design and evaluation of novel therapeutic strategies to overcome GBM-mediated immunosuppression. However, many of the existing models do not adequately recapitulate the tumor microenvironment [2].

Glioma-261 (GL261) is a carcinogen-induced murine glioma cell line initially generated by implanting methylcholanthrene pellets into mouse brains [3]. When engrafted intracranially into syngeneic mice, GL261-derived tumors demonstrate nuclear pleomorphism, mitotic figures, neovascularization, and pseudopalisading necrosis typical of GBM [4]. GL261 cells can be implanted into immunocompetent C57BL/6 mice, and, in contrast to GBM xenograft models, GL261 tumors demonstrate notable invasion into adjacent normal brain [5]. Furthermore, GL261 orthotopic tumors have been shown to be immunosuppressive [6], with GL261 cells secreting TGF-β, and GL261 tumors containing regulatory T-cell infiltration [7,8]. GL261 intracranial growth in C57BL/6 mice is highly reproducible with respect to length of host mouse survival [9] and, as a result, the GL261-C57BL/6 model has been repeatedly used to study immunotherapeutic strategies [10,11].

Transduction of tumor cells with lentivirus expressing firefly luciferase allows for non-invasive, serial monitoring of tumor growth and response to treatment in living animal subjects [12]. Our primary goal for the present study was to evaluate the effect of lentiviral infection of GL261 cells, for expression of firefly luciferase, on immunomodulatory cytokine expression and for tumor growth *in vivo*.

## Methods

### Cell culture, viral transduction, and sample preparation

GL261 murine glioma cells (NCI Tumor Repository, Frederick, MD) were grown and passaged in RPMI-1640 medium containing 10% fetal bovine serum (FBS) and 1% penicillin-streptomycin in a humidified atmosphere of 5% CO_2_. GL261 cells were transduced with HIV1-based lentiviral vector plasmid pHRSIN-CSGW-DINotI expressing firefly luciferase (luc) under the control of the spleen focus-forming virus promoter. Lentiviral vectors were generated by transfection of 293 T cells with plasmids encoding the vesicular stomatitis virus G envelope, gag-pol, and luc (generously provided by Dr. Y. Ikeda, Mayo Clinic, Rochester, MN) [13]. Conditioned medium containing viral vectors were harvested 48 hours post-transfection, filtered (0.45 μm), and frozen until use. GL261 cells were transduced using viral supernatants. Expression of luc was confirmed by measuring luciferase activity (IVIS Lumina imaging station, Caliper Life Sciences), which we have previously demonstrated is highly correlated with cell number [14]. The transduced cell lines will now be referred to as GL261.luc. RNA was extracted using the RNeasy mini protocol (QIAGEN). RNA concentration was determined by spectrophotometry.

### Proliferation assays

For analysis of *in vitro* proliferation, GL261 and GL261.luc cells were transferred to a 96 well plate in quintuplicate at a density of 5,000 cells per well. Proliferation was assessed using the ATPlite Luminescence ATP Detection Assay System (PerkinElmer) at days 1, 2, 4, and 7. To confirm sustained luciferase expression over the relative time course of the *in vivo* study, GL261, GL261.luc and U87.luc cells were transferred to a 24 well plate at a density of 100,000 cells per well and luminescence signals were measured by microplate reader (Tecan Safire^2^) at days 7, 14, and 21. Fold increase was determined by comparing the change in luminescence to that of day 0. Each experiment was repeated in triplicate. All results were verified using a hemocytometer to determine cell count.

### Real-time PCR array

RNA was extracted from GL261 and GL261.luc cells and 2 μg were reverse transcribed (RT2 First Strand, Cat# PAMM-150Z, QIAGEN). Quantitative real-time PCR was used to profile the expression of 82 genes encoding inflammatory mouse cytokines and chemokines according to the manufacturer’s instructions (RT2 Profiler PCR Array, QIAGEN). Each experiment was performed in triplicate.

### Invasion and migration assayss

To assess the invasive capacity of tumor cells, we utilized BD BioCoat Matrigel invasion chambers (BD Biosciences). GL261 and GL261.luc cells were transferred to the invasion chamber using the Cellstripper non-enzymatic cell dissociation solution (Corning). Each cell line was plated in triplicate. Prior to use, chambers were rehydrated with RPMI-1640 for 2 hours at 37°C then plated at a density of 5 × 10^4^ cells per well. After 12 hours of invasion through Matrigel towards a 5% FBS gradient, invasion chambers were fixed in 4% paraformaldehyde for 15 minutes, stained with crystal violet dye, and washed in PBS. Cells were counted using the AMG EVOS XL microscope. 10 high-powered fields (HPF) were counted per well and averaged. Migration was assessed in a similar fashion, except without Matrigel placement in the chambers.

### Intracranial tumor growth

For tumor cell implantation, mice were anesthetized using a ketamine/xylazine mixture. A one cm incision was made over the parieto-occipital bone, and bregma was identified. A sterile 25-gauge sharp needle was used to puncture the skull 2 mm right lateral of bregma and 1 mm anterior to the coronal suture. Cells were injected as previously described by Ozawa et al. at a density of 3 × 10^5^ cells/3 μl [15]. In vivo quantitative bioluminescence imaging was conducted biweekly using the IVIS Lumina imaging station (Caliper Life Sciences). In preparation for imaging, mice were simultaneously anesthetized with ketamine/xylazine and administered with luciferin (D-Luciferin potassium salt, 150 mg/kg, Caliper Life Sciences) via intraperitoneal injection, with mice imaged 12 minutes after injection. Regions of interest encompassing the intracranial area of signal were defined using Living Image software and the total photons/s/sr/cm^2^ (photons per second per steradian per square cm) were recorded. Mice were observed daily until they reached a moribund state, at which time they were euthanized and their brains removed and processed for histopathologic analysis. All animal procedures were approved by the University of California, San Francisco Institutional Animal Care and Use Committee.

### Immunohistochemistry

Sections were cut to 4-μm thickness, deparaffinized in xylene, and rehydrated through graded alcohols to deionized water. Antigen retrieval and endogenous peroxidase block was carried out by standard operating procedure. Immunohistochemistry (IHC) examination of GL261 tumor cells was performed on FFPE sections using the Dako Mouse EnVisionTM + HRP method. The following antibodies are used for detection of (i) Ki-67 [monoclonal rat anti-mouse Ki-67 antigen, clone TEC-3 (1:31250) (DAKO M7249) DAKO, Glostrup, Denmark] (ii) CD31 [goat polyclonal anti-mouse CD31 antigen, clone M-20 (1:1000) (sc-1506) Santa Cruz Biotechnology, Dallas, Texas U.S.A] and (iii) CD3 [Rabbit Polyclonal anti-mouse CD3 antigen, clone F7.2.38 (1:200) (Dako A0452) DAKO, Glostrup, Denmark] using DAKO auto-stainer.

### Biomarker evaluation

The slides were digitized using NanoZoomer 2.0-HT: C9600-13 scanner (Hamamatsu Photonics, Iwata City, Japan) at 20X magnification. For Ki-67, CD31, and CD3 staining, six peripheral/ invasive tumor edge regions were sampled with a fixed field of view of 0.427 mm^2^ per immunostained slide [16]. Areas of necrosis were discarded during the evaluation and the senior histopathologist (VP) was blinded to the experimental treatment. ImmunoRatio software was used to quantify Ki67 and CD3 immunoreactivity, and CD31 positively stained endothelial capillaries were manually enumerated. The Ki-67 Labeling Index (LI) was calculated as a percentage of positively stained cells per the total number of tumor cells considered for evaluation.

### Statistical analysis

Differences in continuous variables between two groups were compared by Student’s *t*-test. Kaplan-Meier estimates were generated to illustrate the overall survival curves. Animal deaths not related to tumor growth were censored. Differences in overall survival between groups were compared by the log-rank test. All analyses were carried out using PASW Statistics 18 (SPSS, Inc). *P*-values less than 0.05 were considered statistically significant.

## Results

### Luciferase expression does not affect in vitro proliferation

**Figure 1:**
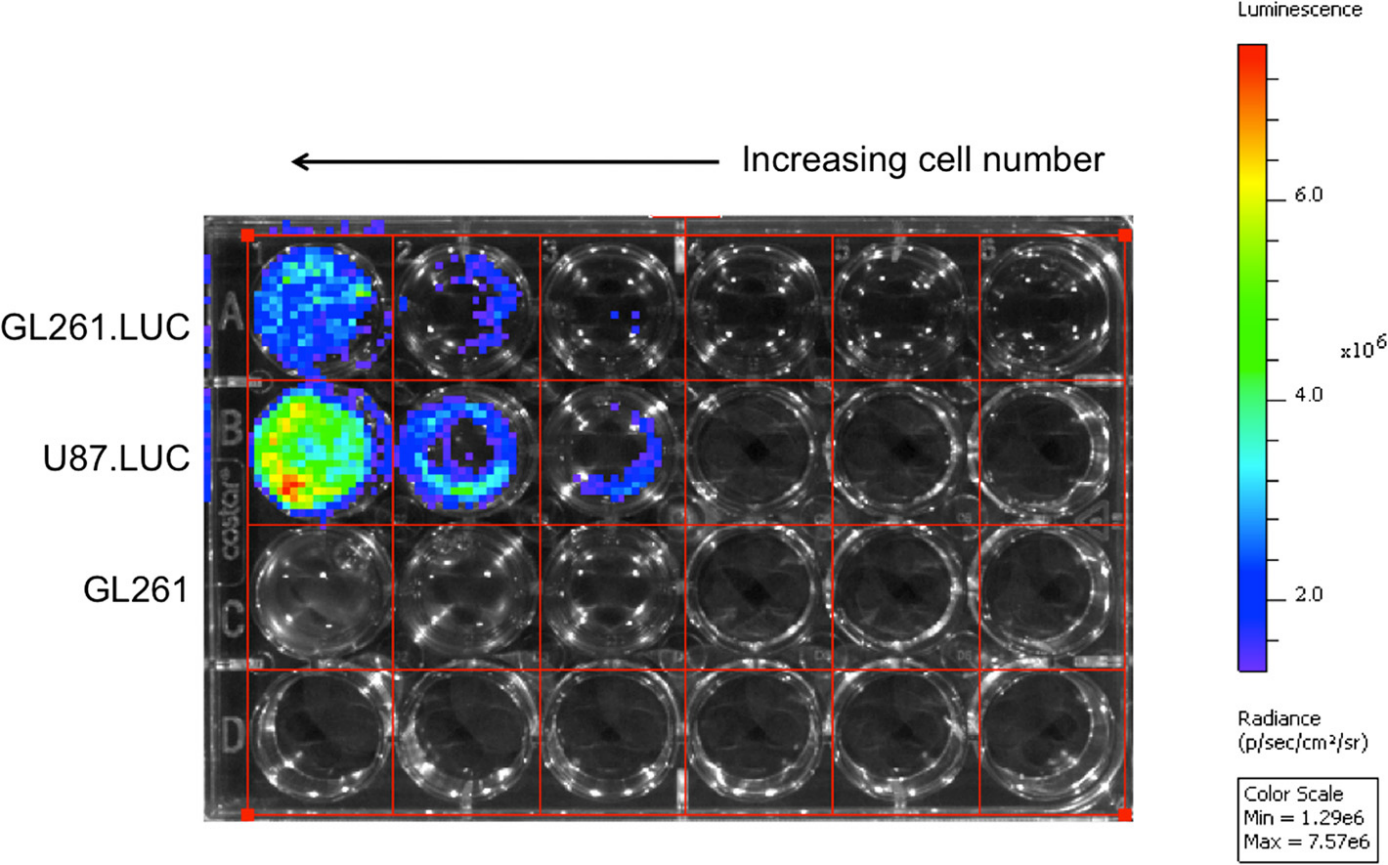
**Luciferase activity in GL261.luc cells.** U87MG cells, previously modified with the same lentivirus used here for GL261 modification [14], were used as a control for assessing luciferase activity in transduced GL261 cells. U87MG.luc cells were plated at densities of 1x10^6^, 0.5x10^6^, 0.25x10^6^, 0.1x10^6^, 0.05x10^6^, and 0.025x10^6^ cells/well, from left to right. GL261 (negative control) and GL261.luc cells were plated at densities of 1x10^6^, 0.5x10^6^, and 0.25x10^6^ cells/well.

**Figure 2:**
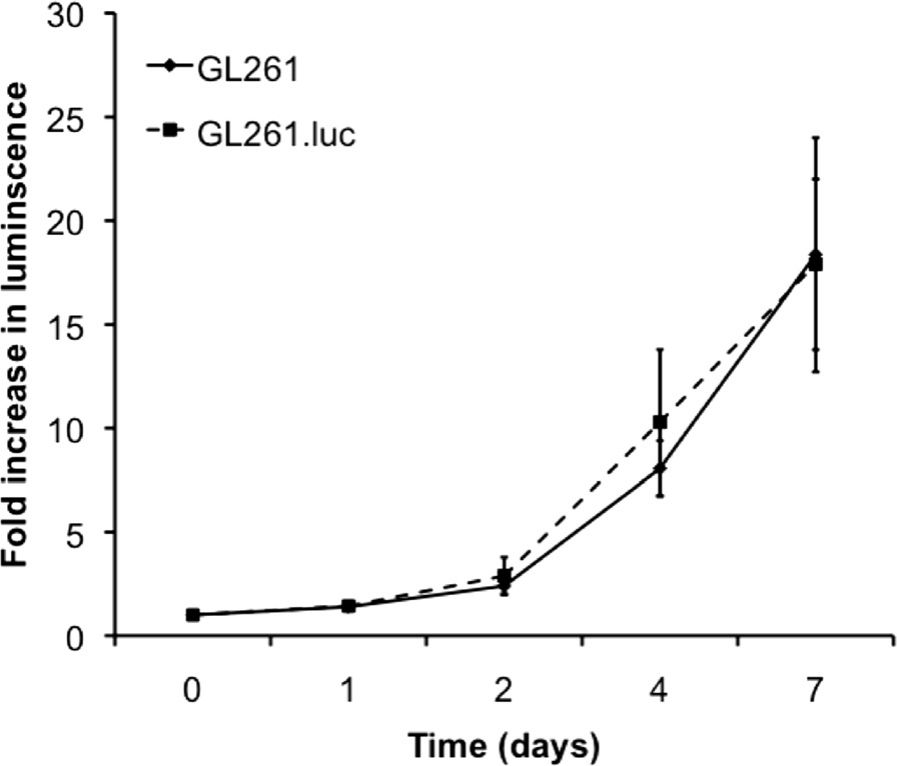
**Luciferase expression does not affect proliferation Luciferase expression by GL261.luc cells does not cause a difference in proliferation,** ***in vitro*** **, as demonstrated by ATP-based viability assay.** The graph shows fold increase, relative to day 0, as determined by comparing the luminescence at the specified time point to the luminescence at time 0.

GL261 cells were infected with luciferase containing lentivirus (referred to as GL261.luc cells), and maintained in culture using *in vitro* growth conditions identical to that for unmodified GL261. Several weeks after infection, GL261.luc cells were evaluated for luciferase expression, with moderate luciferase activity detected (Figure 1). Cells were then compared for *in vitro* growth rate, using an ATP-based viability assay. Numbers of viable cells in GL261 and GL261.luc cultures were compared at day 0, 1, 2, 4, and 7, after plating. This analysis revealed no significant difference in viable cells at any time point (*P* > 0.05 at all time points, Figure 2).

### Luciferase expression does not markedly change cytokine expression

**Figure 3:**
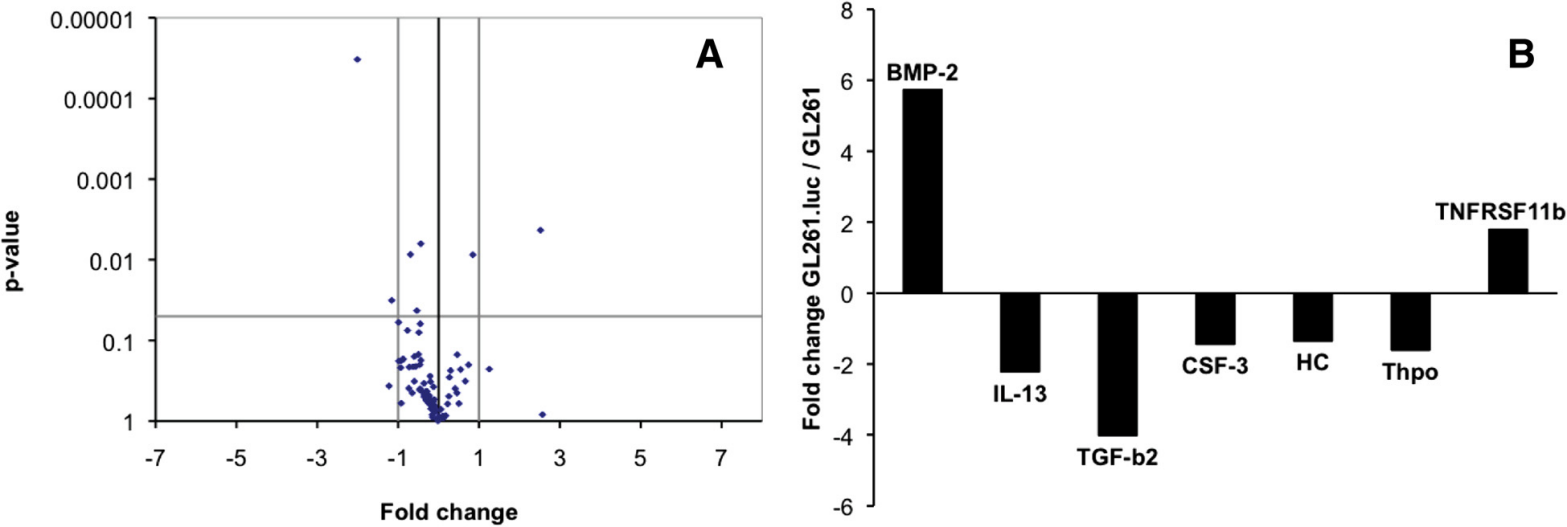
**Differences in gene expression between GL261 and GL261.luc.** Volcano plot **(A)** demonstrating the differences in gene expression, for 82 cytokine and chemokine genes, between GL261.luc and unmodified GL261 cells. The horizontal grey line indicates the level at which a significance difference (*P* = 0.05) exists. Points above that line represent genes whose expression is significantly different between the two cells. The vertical grey lines indicate a fold change of 2: either increased or decreased by a factor of 2 in GL261.luc cells. Points outside of the vertical lines represent genes which are more than 2-fold changed. A bar graph **(B)** depicts the extent of change for the seven genes whose expression is significantly different between GL261.luc and GL261.

We next evaluated the effect of luciferase expression on GL261 immunomodulatory cytokine and chemokine expression. For this, we used RT-PCR to determine the mRNA expression level for 82 genes, with results showing statistically significant differences between GL261 and GL261.luc cells in seven instances (Figure 3A,B). Of these, the expression difference was greatest for BMP-2 (5.74-fold increased in GL261.luc), IL-13 (2.23-fold decreased in GL261.luc), and TGF-β2 (4.03-fold decreased in GL261.luc). All other expression changes were less than two-fold.

### Luciferase expression does not affect GL261 in vitro invasion or migration

**Figure 4:**
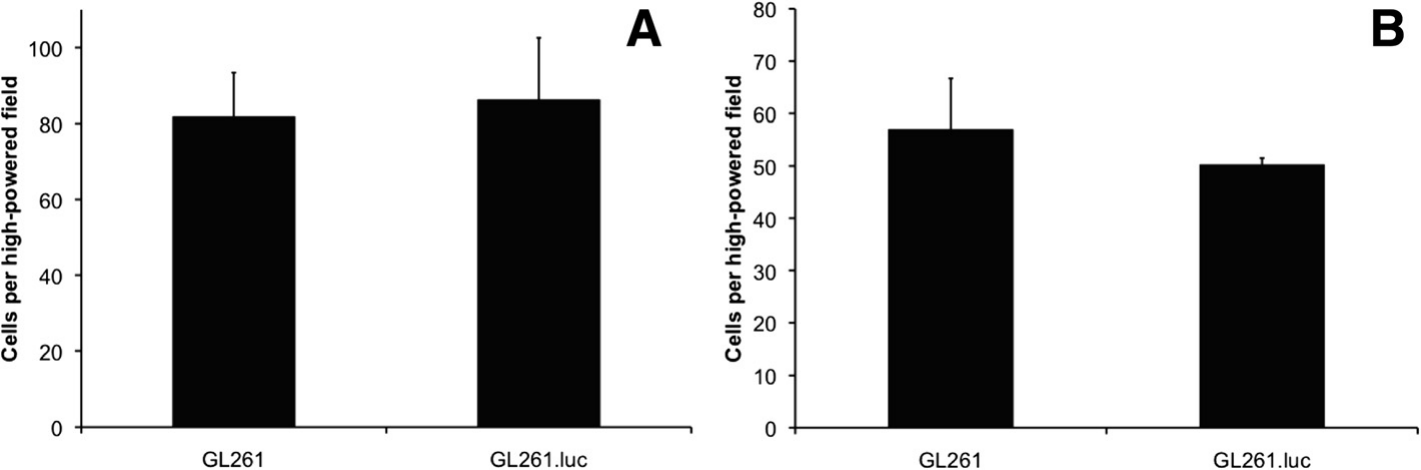
**Luciferase expression does not alter invasion or migration.** The bar graphs showing no difference in *in vitro* invasion **(A)** and migration **(B)** between GL261.luc and GL261 cells. Error bars indicate standard error between triplicates.

We next compared the *in vitro* migration and invasion of GL261 vs. GL261.luc cells. At 12 hours post seeding in modified Boyden chambers, an average of eighty-two GL261 cells/HPF had invaded through Matrigel coated membranes, compared to eighty-six GL261.luc cells/HPF (Figure 4A, *P* = 0.67). Similar results were obtained when examining cell migration though a porous membrane (Figure 4B, GL261, 57 cell/HPF; GL261.luc, 50 cells/HPF; *P* = 0.26).

### Luciferase expression does not affect overall survival

**Figure 5:**
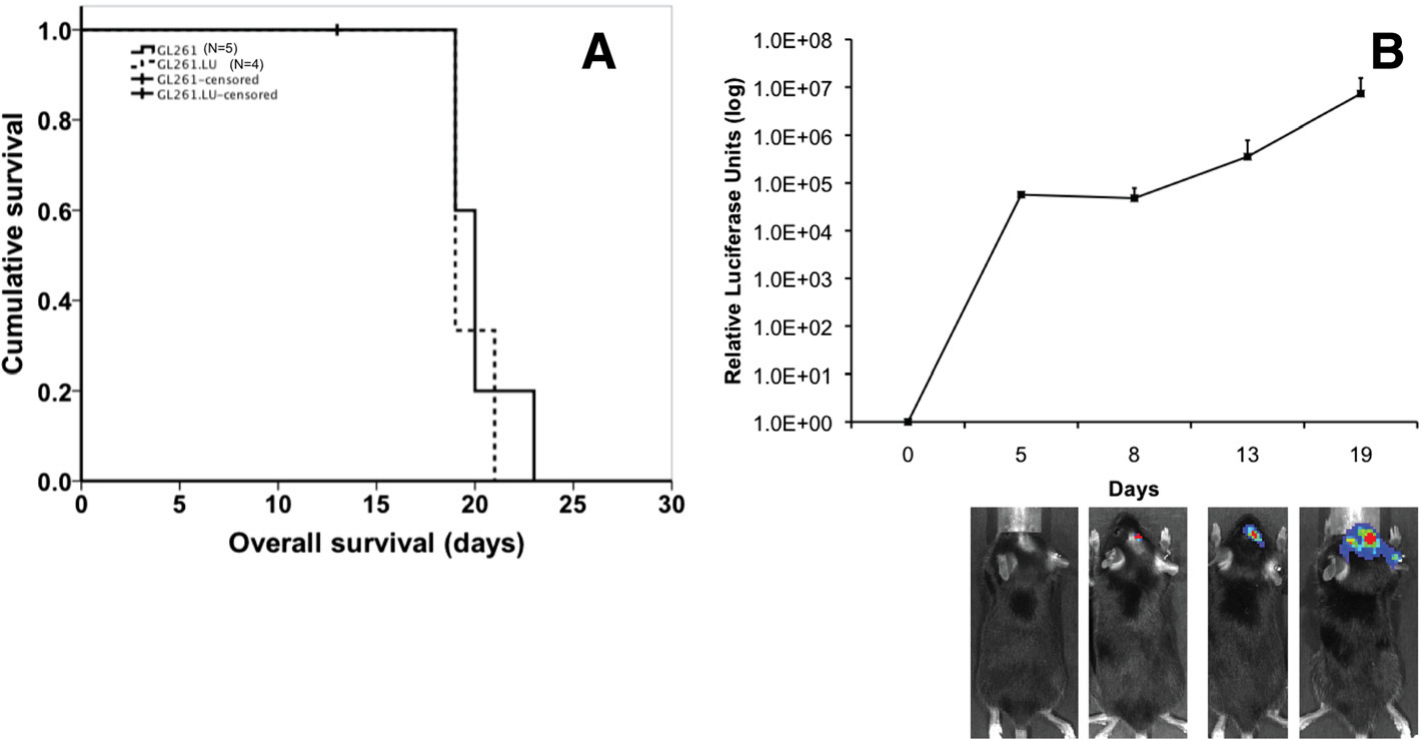
**Luciferase expression does not affect overall survival.** Kaplan-Meier survival analysis **(A)** demonstrates no difference in overall survival for mice implanted intracranially with either GL261.luc (dashed line) or GL261 cells (solid line). One animal in the GL261.luc group was censored due to procedural-related death. *In vivo* quantitative bioluminescence imaging **(B)** demonstrates non-invasive monitoring of intracranial tumor growth in animals implanted with GL261.luc cells.

**Figure 6:**
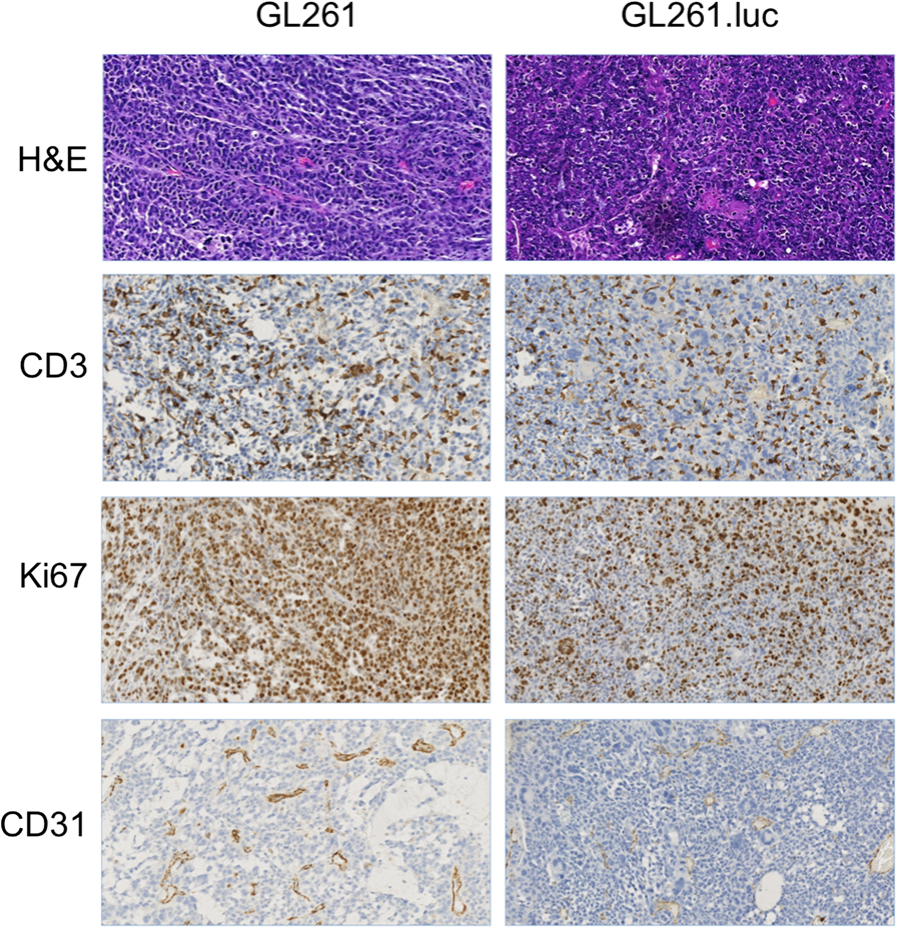
**Histopatholgic and immunohistochemical analysis of tumor specimens from GL261 and GL261.luc bearing animals.** Hematoxylin and Eosin (H&E) staining was used for conventional morphologic analysis of tumor. Ki-67 staining was for examining tumor cell proliferation, whereas C3 and CD31 staining was for T-cell infiltration and tumor neovascularization, respectively. Magnification is 200 X.

**Figure 7:**
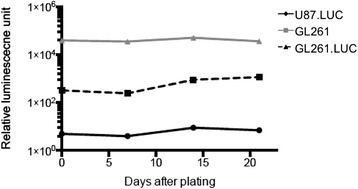
***In vitro*** **luciferase expression of GL261.luc over 21 days.** GL261.luc cells were maintained in culture over 21 days. At day 0, 7, 14, and 21, cells were seeded in 6-well plates at a defined density and were evaluated for bioluminescence. GL261 and U87.luc cells were used as negative and positive controls, respectively.

A critical characteristic of an orthtopic glioma engraftment model is the reproducible establishment of tumors from implanted cells. To investigate the effect of luciferase modification on GL261 cell tumorigenicity, we implanted GL261 and GL261.luc cells into the brains of C57BL/6 mice. In the GL261.luc group, there was one accidental death, which was censored for survival analysis. Median overall survival was 20.2 days for GL261-bearing animals and 19.7 for GL261.luc-bearing animals (Figure 5A, *P* = 0.62), with no survival shorter than 19 days nor longer than 23 days for either cohort of mice. Gross, macroscopic analysis of the brain for each injected mouse showed readily observable tumor. Luciferase activity of intracranially injected GL261.luc cells was detectable by 5 days post-implantation, with steadily increasing tumor luminescence evident days 8, 13 and 19 post-implantation (Figure 5B). Histologically, there was no difference in appearance of the GL261 tumors compared to GL261.luc (Figure 6). Likewise, there was no significant difference in CD3, Ki67, or CD31 staining between intracranial GL261 and GL261.luc tumors (Table 1, Figure 6). To confirm that there was no change in luciferase expression by GL261.luc over the relative time course of the intracranial experiment, we examined a defined number of GL261.luc cells for changes luciferase activity over 21 days. As expected based on the results in Figure 1, GL261.luc demonstrates less robust luciferase activity compared to U87.luc, however luminescence activity of each cell line does not exhibit much change over the course of the timeframe of our intracranial experiment (Figure 7).

**Table 1:** **Luciferase expression does not significantly impact histopathologic characterization of GL261 cells**

**Marker**	**GL261 N = 5**	**GL261.luc N = 4**	***P*** **-value**
CD3*	31.3	23.7	0.24
Ki67^§^	67.3	48.3	0.18
CD31*	31.7	18.3	0.07

*Cells per high power field, ^§^Percentage positive cells.

Analysis of CD3, Ki67, and CD31 in tumor harvested from GL261 and GL261.luc bearing animals. For each marker, six random areas were counted and averaged per tumor specimen.

## Discussion

In this study we have examined the effect of lentiviral transduction and expression of firefly luciferase on murine glioma cell biological properties, both *in vitro* and *in vivo*. Among the issues we sought to address was whether luciferase expression alters syngeneic host mouse response to tumor, which, if realized, would detract from the benefit of developing derivative cells for *in vivo* imaging. To achieve our study goals, we generated murine GL261 cells with stable expression of the luciferase gene. Analysis of parental and luciferase-modified cells *in vitro* revealed that luciferase expression resulted in no significant effect on the expression of 75 cytokine and chemokine genes, from 82 genes total that were examined. Gene expressions most effected by the luciferase modification were BMP-2, up-regulated approximately six-fold in luciferase-modified cells, IL-13, and TGF-β2, with these latter two genes down-regulated in luciferase-modified cells by more than 2- and 4-fold, respectively.

With respect to potential consequences of these gene expression changes, BMP-2 has been shown to induce differentiation of glioma stem-like cells and, thus, modulate chemosensitivity *in vitro* [17]. This luciferase modification-associated change, therefore, should be taken into consideration with future studies using the GL261.luc model for testing chemotherapeutic agents. Less is known about the role of IL-13 in glioblastoma biology. A recent clinical study demonstrated that changes in plasma IL-13 levels, during patient treatment with a novel anti-angiogenic agent, were associated with increased on-target toxicities [18].

Significantly more is known about the role of TGF-β in GBM biology. In addition to promoting glioma cell proliferation, angiogenesis, and invasion, TGF-β enhances glioma cell immune evasion [19]. Pharmacological inhibition of TGF-β in animal models of glioma can induce tumor rejection and increase survival, with associated increases in tumor immune cell infiltration [20,21].

The identification of significant gene expression changes in 8.5% of the genes surveyed suggests the need for a more detailed analysis of transcriptome effects from lentiviral luciferase modification, and further characterization of GL261.luc will likely include the use of gene expression arrays for a comprehensive analysis of transcriptional changes. However, and despite the luciferase-associated change in expression for a minor fraction of the genes surveyed, these changes had no significant effect on GL261 biologic properties. GL261.luc invasion and migration, *in vitro*, was essentially indistinguishable from unmodified GL261 cells, and, importantly, animals implanted intracranially with GL261.luc cells had lengths of survival that were highly similar to mice receiving intracranial injection of unmodified GL261 cells.

Likewise, there were no differences in T-cell infiltration into GL261 vs. GL261.luc tumors, as indicated by similar numbers of CD3 positive cells, and suggesting that cytokine expression differences, noted above, do not have discernable effect on host immune response.

The current study did not include serial monitoring of unmodified GL261 tumor growth. Non-invasive CT or MR imaging, could be used for this purpose, although the basis for doing so is not compelling, given the number of similarities in biologic properties, between unmodified and luciferase-modified GL261, we have identified. Furthermore, in a recent study by Huang *et al*. evaluated the growth of GL261.luc tumors in PDGF knockout C57BL/6 mice [22]. As would be expected, the growth and survival curves demonstrated are very similar to those in the present study. On day 15 post implantation, the researchers performed contrast enhanced MRI of the mice and demonstrated good correlation between MRI volumes and bioluminescence. Of note, these results are in contrast to luciferase modified U87 cells previously reported by our group, which demonstrate a much higher range of luciferase activity [14]. This apparent inconsistency is to be expected based on the data presented in the current study which show lower baseline luciferase activity for GL261.luc compared to U87.luc.

## Conclusions

GL261 engrafted tumors recapitulate several critical characteristics of human GBM. Lentiviral transduction with the luciferase gene results in stable luciferase expression, and allows for non-invasive imaging of tumor growth. Luciferase expression may be associated with change in the expression of a small subset of cytokine genes, although this does not result in altered biologic properties of GL261 tumor cells, or in tumors established from GL261 cells.

## Abbreviations

GBM: Glioblastoma
GL261: Glioma-261
FBS: Fetal bovine serum
luc: Luciferase
HPF: High-powered fields
IHC: Immunohistochemistry
LI: Labeling Index
